# H3K27m3 overexpression as a new, BCL2 independent diagnostic tool in follicular and cutaneous follicle center lymphomas

**DOI:** 10.1007/s00428-022-03347-y

**Published:** 2022-06-04

**Authors:** Magdalena M. Brune, Visar Vela, Ivana Bratic Hench, Susanne Dertinger, Vanessa Borgmann, Stefan Dirnhofer, Alexandar Tzankov

**Affiliations:** 1 Institute of Medical Genetics and Pathology, University Hospital Basel, University Basel, Schönbeinstrasse 40, CH-4031 Basel, Switzerland; 2 Institute of Pathology, Hospital Feldkirch, Feldkirch, Austria; 3 Institute of Pathology and Neuropathology, Eberhard Karls University of Tübingen and Comprehensive Cancer Center, University Hospital Tübingen, Tübingen, Germany

**Keywords:** Follicular lymphoma, Follicular hyperplasia, H3K27m3, *EZH2*, BCL2

## Abstract

Approximately 15% of follicular lymphomas (FL) lack overexpression of BCL2 and the underlying translocation t(14;18). These cases can be diagnostically challenging, especially regarding follicular hyperplasia (FH). In a subset of FL, mutations in genes encoding for epigenetic modifiers, such as the histone-lysine N-methyltransferase *EZH2* (enhancer of zeste homolog 2), were found, which might be used diagnostically. These molecular alterations can lead to an increased tri-methylation of histone H3 at position lysine 27 (H3K27m3) that, in turn, can be visualized immunohistochemically. The aim of this study was to analyze the expression of H3K27m3 in FL, primary cutaneous follicle center lymphomas (PCFCL), and pediatric-type FL (PTFL) in order to investigate its value in the differential diagnosis to FH and other B cell lymphomas and to correlate it to BCL2 expression and the presence of t(14;18). Additionally, the mutational profile of selected cases was considered to address H3K27m3’s potential use as a surrogate parameter for mutations in genes encoding for epigenetic modifiers. Eighty-nine percent of FL and 100% of PCFCL cases overexpressed H3K27m3, independently of BCL2, EZH2, and the presence of mutations. In contrast, 95% of FH and 100% of PTFL cases lacked H3K27m3 overexpression. Other B cell lymphomas considered for differential diagnosis also showed overexpression of H3K27m3 in the majority of cases. In summary, overexpression of H3K27m3 can serve as a new, BCL2 independent marker in the differential diagnosis of FL and PCFCL, but not PTFL, to FH, while being not of help in the differential diagnosis of FL to other B cell lymphomas.

**Supplementary Information:**

The online version contains supplementary material available at 10.1007/s00428-022-03347-y.

## Introduction

Follicular lymphoma (FL) is a germinal center–derived B cell malignancy accounting for approximately 15% of all lymphomas worldwide, with the highest incidence rates in the USA and Western Europe. It occurs predominantly in the elderly and seems to be associated with exposure to pesticides and herbicides [1, 2]. Its characteristic hallmark translocation t(14;18) leads to an overexpression of the BCL2 protein, which provides the B cell with an evolutionary advantage in the lymph node, rescuing B cells with weak B cell receptor affinities that would otherwise undergo apoptosis. The emergence of the t(14;18) is regarded as the first oncogenic event in the development of FL, although, taken by itself, it most probably has not the potential to induce lymphomagenesis, as naïve t(14;18)-positive circulating B cells can be observed in healthy elderly individuals and additional genetic hits seem to be necessary for disease induction [1]. Furthermore, about 15% of FL lack this translocation and constitute a genetically heterogeneous group [3], which renders them diagnostically difficult, especially in the differential diagnosis to reactive follicular hyperplasia (FH).

Apart from t(14;18), recurrent mutations in epigenetic regulators are identified in more than two-thirds of investigated FL cases [4]. One mechanism of epigenetic remodeling is the methylation of histones, which is catalyzed, among others, by the evolutionary highly conserved polycomb repressive complex 2 (PRC2). One of the functional subunits of PRC2 is the histone-lysine N-methyltransferase enzyme *EZH2* (enhancer of zeste homolog 2) that is responsible for the methylation of histone 3 (H3) at the position lysine 27 (H3K27) [5], which, in turn, is functionally associated with gene repression executed by H3 [6]. Consequently, *EZH2* has a great impact on gene expression and, specifically, on germinal center formation [7]. Thus, alterations of *EZH2* may exhibit tremendous effects on the germinal center machinery. In 2010, recurrent somatic mutations affecting the SET domain of *EZH2 (Y641)* have been identified in 22% of germinal center–derived diffuse large B cell lymphomas (DLBCL, GCB) and 7% of FL [8]. In the following years, recurrent mutations in *EZH2* have been described in up to 29% of FL [9]. Most interestingly, all of the investigated cases in the study of 2010 displayed a heterozygous expression of both, wild-type and mutant alleles of *EZH2* [8]. Sneeringer et al. were able to show that both alleles were needed to induce a disease-associated enzymatic malfunction: while the wild-type allele has its greatest catalytic effect in the mono-methylation of H3K27 (H3K27m1), the mutant allele of *EZH2* has an augmented catalytic efficacy in the di- and tri-methylation step, but only very limited catalytic capacity at mono-methylation [10]. Thus, only the coordinated interaction between the wild-type and the mutant *EZH2* allele can lead to an increased tri-methylation of H3K27 (H3K27m3), which is associated with lymphomagenesis [11]. In 2012, a second somatic mutation in *EZH2* was found (*A677*), which has also been linked to aberrant H3K27 tri-methylation [12]. Later, it could be demonstrated that the specific inhibition of the *EZH2*’s methyltransferase activity by highly selective small molecules leads to a decrease of H3K27m3 levels and the reactivation of silenced genes [13]. This paved the way for the development of *EZH2*-targeted therapies that by now show promising results in ongoing trials in refractory or relapsed FL [14, 15]. A reliable, inexpensive, and ubiquitous available surrogate read-out-marker for the *EZH2*-status might therefore be desirable, helping to minimize molecular testing, which is more complex, expensive, and not always at hand. One possibility would be the immunohistochemical visualization of H3K27m3 or the application of anti-EZH2 antibodies [16]. Unfortunately, the latter never came into widespread use, because there is no existing mutation-specific antibody on the market and, as stated, cooperation of both mutant and wild-type alleles is needed for exertion of the oncogenic potential of *EZH2.*

So far, contradictory results are available concerning H3K27m3’s or EZH2’s use as surrogate markers [17, 18], with a single study confirming H3K27m3’s feasibility in FL [9]. Further on, H3K27m3 expression has never been used for diagnostic purposes in the differential diagnosis of FL to FH and other B cell lymphomas. For that reason, we investigated the expression pattern of H3K27m3 and EZH2 in 148 FL cases (daily routine excisional biopsies and tissue micro-arrayed cases) of all grades, both BCL2-positive and BCL2-negative as well as *BCL2*-rearranged and not-rearranged, in 9 primary cutaneous follicle center lymphomas (PCFCL) and 5 pediatric-type FL (PTFL) and compared the staining results to a control cohort of various reactive conditions and different B cell lymphomas. Finally, with the help of selected cases, we examined the correlation of immunohistochemical expression of H3K27m3 with the genetic profile, with special regard to the presence of mutations in *EZH2*.

## Material and methods

### Cohort

We compiled a cohort of 266 cases of different mature B cell lymphomas and benign reactive conditions, either micro-arrayed [19–22] or available as conventional excisional biopsies originating from the archives of the Institute of Pathology, University Hospital Basel, Switzerland and the Institute of Pathology and Neuropathology, University Hospital Tübingen, Germany. The study design was approved by the Ethics Committee of the University Hospital Basel (EKNZ-2014–252). All cases were re-examined by two pathologists (AT and MMB) and stained for H3K27m3, as well as FL and PCFCL were also stained for (at least) one BCL2 clone (SP66, E17, or 124) and EZH2 on an automated immunostainer (Benchmark Ultra from Roche/Ventana). The 5 PTFL were part of another study [23] and due to material exhaustion, only single slides for the H3K27m3 staining were available. Details of the applied antibodies, dilutions and antibody retrieval, and incubation can be found in Suppl. Table 1.

Details of the complete cohort are given in Table 1. The FL cohort contained 121/148 (82%) low-grade (G1/G2) FL, 14/148 (9%) grade 3A FL, 3/148 (2%) grade 3B FL, and 10/148 (7%) grade 3 FL, which were not further classifiable as either A or B.

**Table 1:** Details on H3K27m3 and EZH2 expression of the whole cohort

	H3K27m3		EZH2				
	No or weak expression	Over-expression	0 negative	1 weak	2 moderate		3 strong
FL	11% (15/135)	89% (120/135)	34% (31/91)	43% (39/91)	22% (20/91)		1% (1/91)
PCFCL	0% (0/9)	100% (9/9)	Not evaluated				
PTFL	100% (5/5)	0% (0/5)	Not evaluated				
MZL	12% (2/17)	88% (15/17)	Not evaluated				
CLL/SLL	4% (1/28)	96% (27/28)	25% (5/20)	50% (10/20)	20% (4/20)	5% (1/20)	
MCL	0% (0/14)	100% (14/14)	0% (0/1)	0% (0/1)	100% (1/1)	0% (0/1)	
FH	95% (40/42)	5% (2/42)	0% (0/11)	0% (0/11)	46% (5/11)	55% (6/11)	
IgG4 LA	100% (2/2)	0% (0/2)	Not evaluated				
HVCD	100% (1/1)	0% (0/1)	Not evaluated				

*FL*, follicular lymphoma; *PCFCL*, primary cutaneous follicle center lymphoma; *PTFL*, pediatric-type follicular lymphoma; *MZL*, nodal marginal zone lymphoma; *CLL/SLL*, chronic lymphocytic B cell leukemia/small lymphocytic B cell lymphoma; *MCL*, mantle cell lymphoma; *FH*, follicular hyperplasia; *IgG4 LA*, IgG4 associated lymphadenopathies with follicular hyperplasia; *HVCD*, hyaline-vascular Castleman’s disease

Selected cases of FL (14/148) and FH (3/42) underwent molecular work-up with our customized lymphoma panel as described elsewhere [24]. Only somatic mutations described as pathogenic or possible/probable pathogenic were considered in further analysis.

### Interpretative immunohistochemical examination

Stainings for BCL2 were either interpreted as negative, if there was no detectable expression in the neoplastic germinal center B cell-equivalents, or as positive, if there was an expression in these cells with at least one of the applied antibody clones (SP66, E17, or 124).

**Fig. 1:**
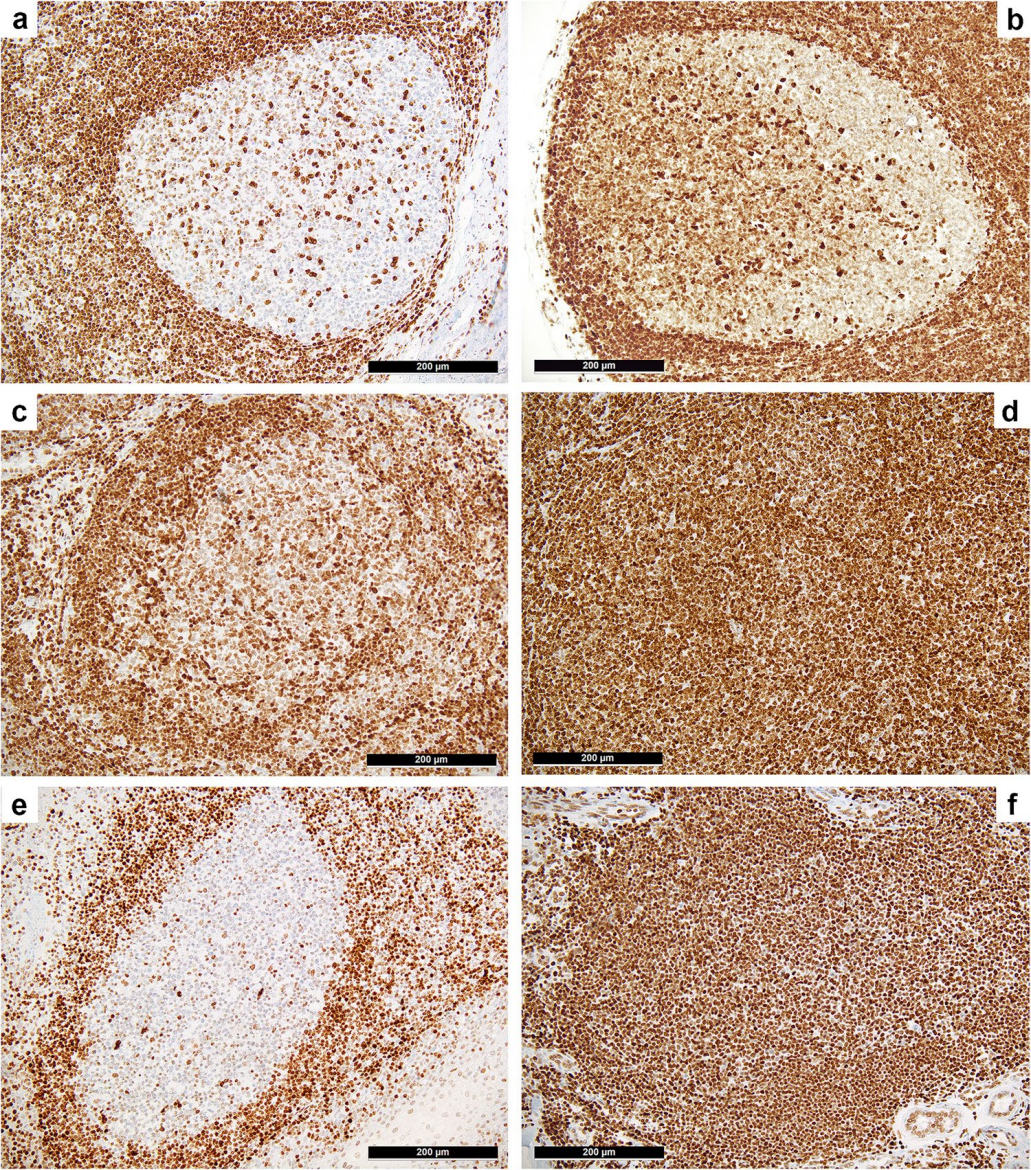
*H3K27m3 expression in physiologic and neoplastic germinal centers*. “Physiologic-type” expression of H3K27m3 in germinal centers of follicular hyperplasia (FH) with single cell positivity of centroblasts and follicular T-helper cells (**a**), occasionally with a clearly recognizable dark/pale zonation (**b**). Moderate overexpression (**c**) of H3K27m3 in a neoplastic germinal center of follicular lymphoma (FL) grade 3B, and a strong and diffuse overexpression in a case of low grade FL (**d**), representing the most commonly observable H3K27m3 staining pattern in FL in general; note the remnants of a mantle zone showing a distinct “onion skinning” in the left upper part of **d**. FH of the skin (**e** with a skin adnexal structure in the right lower corner) showing “physiologic type” H3K27m3 expression that contrasts the strong expression in a neoplastic germinal center of a primary cutaneous follicle center lymphoma (**f**)

Interpretation of H3K27m3 expression in physiologic and neoplastic germinal centers of FH and FL, respectively, was performed as follows: so-called physiologic-type staining results included negative or barely detectable expression in the germinal center as well as physiological, clearly zonated expression with up to moderate staining of centroblasts, while a positive staining result was assigned if there was a moderate or strong overexpression in the (neoplastic) germinal centers without recognizable zonation. Figure 1a-f and Suppl. Figure 1 illustrate the interpretative results for H3K27m3.

**Fig. 2:**
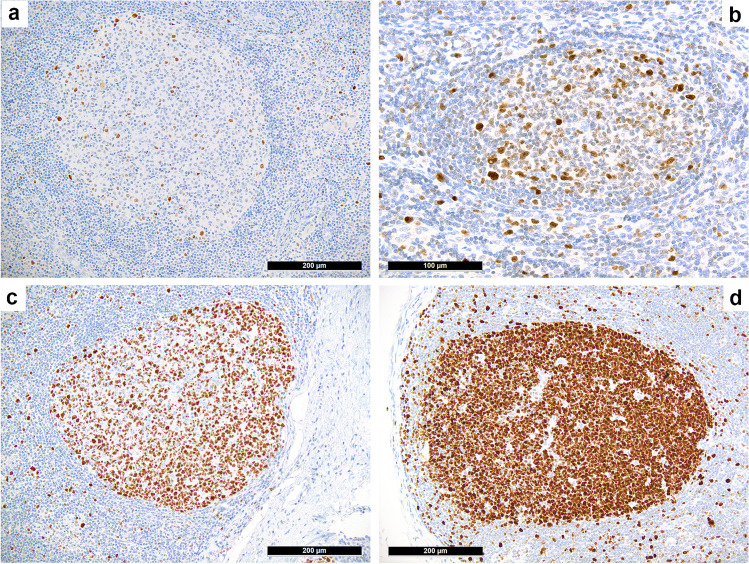
*EZH2 expression in physiologic and neoplastic germinal centers*. Semi-quantitative assessment of EZH2 with barely detectable single cell expression (= 0) in low grade follicular lymphoma (FL) (**a**) and a weak expression (= 1) in FL grade 3A (**b**). Moderate expression (= 2) of EZH2 (**c**) within the same germinal center of follicular hyperplasia (FH) as shown in Fig. 1a and strong expression (= 3) of EZH2 (**d**) within the same germinal center of FH as shown in Fig. 1b

The expression of EZH2 in physiologic and neoplastic germinal centers of FH and FL (Suppl. Figure 2), respectively, was assessed semi-quantitatively: 0, not detectable or single cell positivity; 1, weak and/or partial expression; 2, moderate expression; and 3, strong expression (Fig. 2a-d).

In the remaining cohort, the expression of H3K27m3 and EZH2 respectively, did not refer to the expression in (neoplastic) germinal centers, but to the tumor cells.

### Fluorescence in situ hybridization for BCL2-rearrangements

In 54 (37%) FL cases, the results of fluorescence in situ hybridization (FISH) with a break-apart probe targeting the *BCL2*-locus were available, as this analysis has already been performed in the routine diagnostic work-up, as per standard operating procedures valid between 2015 and 2020 in the accredited lab-part of our institute.

### Statistics

The statistical analysis including descriptive assessment was performed with the SPSS 28.0 software package (Armonk, NY, USA). Crosstabulation and the Fisher’s exact test or the chi-square test were used, as appropriate, to calculate correlations. *P*-values < 0.05 were considered significant whenever possible two-sided tests were applied.

## Results

### BCL2 status of FL and PCFCL cases

The immunohistochemical BCL2 expression of all 148 FL cases was reproduced for purposes of the present study, of which 19/148 (13%) were negative. The remaining 129/148 (87%) were positive by at least one of the applied anti-BCL2 antibodies (SP66, E17, or 124). Forty-one of fifty-four (76%) FL were *BCL2*-rearranged. All FISH-positive cases were immunohistochemically BCL2 positive. Thirteen of fifty-four (24%) cases were *BCL2*-rearrangement negative, and six of them displayed immunohistochemical BCL2 expression. Six of nine (67%) of PCFCL were BCL2 negative, while the other 3 were weakly positive; the two FISH-tested cases were both not rearranged.

### H3K27m3 expression

Detailed staining results of the complete cohort for the expression of H3K27m3 and EZH2 are found in Table 1. Interpretable results of H3K27m3 staining were obtained in 135/148 of FL cases. A total of 120/135 (89%) cases displayed a moderate to strong overexpression of H3K27m3 in the neoplastic germinal centers (Fig. 1c,d), whereas only 15/135 (11%) showed a “physiologic-type” expression of this marker. Fisher’s exact test could not detect a statistical correlation (*P* = 0.423) between the expression of H3K27m3 and BCL2 expression in FL. The same applied to the relationship between H3K27m3 and *BCL2* rearrangements (*P* = 0.655) and the grading (*P* = 0.504). Details and crosstabulation are given in Table 2. Furthermore, H3K27m3 expression did not correlate with the presence of any of the detected mutations in the 14 molecularly analyzed FL cases (Suppl. Table 2).

**Table 2:** Correlations between H3K27m3 and immunohistochemical BCL2 expression (upper) and *BCL2*-rearrangements (lower) in the investigated follicular lymphoma cases

Correlation of H3K27m3 and BCL2 expression	H3K27m3 expression		Correlation of H3K27m3 expression and *BCL2* FISH	H3K27m3 expression	
*n* = 135	Physiologic type expression	Overexpression	*n* = 47	Physiologic type expression	Overexpression
BCL2 IHC positive	12/117	105/117	*BCL2* FISH positive	6/34	28/34
	− 10%	− 90%		− 18%	− 82%
BCL2 IHC negative	3/18	15/18	*BCL2* FISH negative	1/13	12/13
	− 17%	− 83%		− 8%	− 92%
*P*-value	0.423		*P*-value	0.655	

**Fig. 3:**
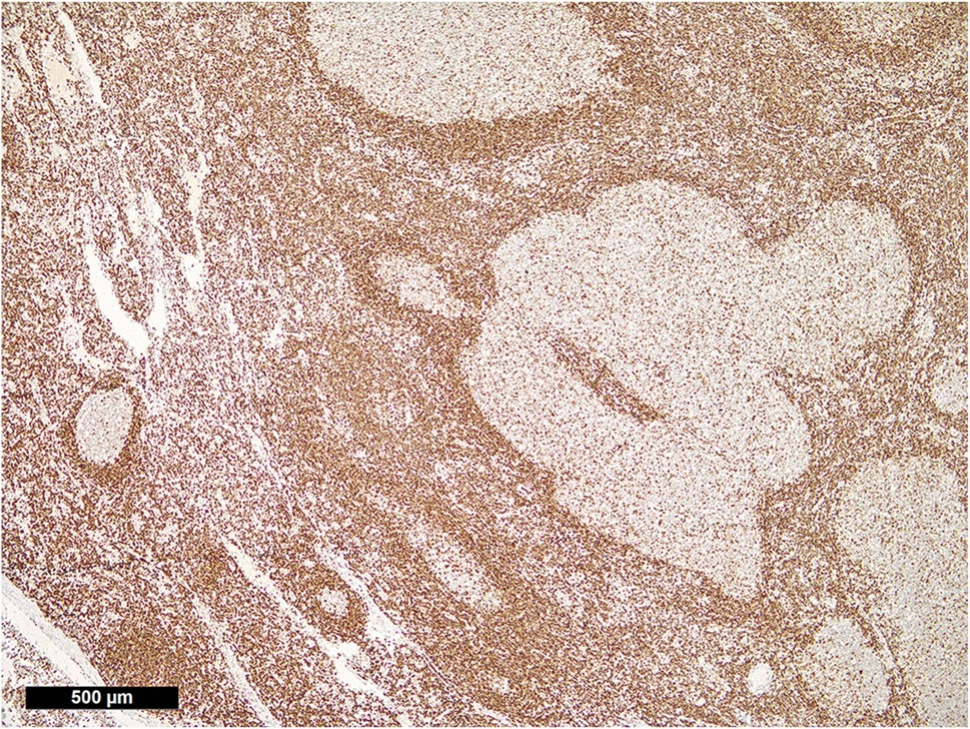
*H3K27m3 expression in pediatric type follicular lymphoma*. Note the “physiologic-type” (comparable with that of a reactive germinal center in the middle of the left border of the microphotograph) weak expression to negativity for H3K27m3 in the neoplastic germinal centers and the staining enhancement at their irregular serpiginous borders

All 9 PCFCL displayed moderate to strong overexpression of H3K27m3 (Fig. 1f), clearly contrasting both the pattern in reactive germinal centers (Fig. 1e) and the immunohistochemical negativity for BCL2 (by the antibody clone SP66) in 6/9 cases and the weak BCL2 expression in the remaining 3 cases. All PTFL showed a “physiologic-type” expression of H3K27m3 (Fig. 3).

Fifteen of seventeen (88%) of the investigated marginal zone lymphomas (MZL) showed an overexpression of H3K27m3, and the staining displayed the pattern of follicular colonization in respective cases (Suppl. Figure 3). Cases of chronic lymphocytic leukemia/small lymphocytic lymphoma (CLL/SLL) and mantle cell lymphoma (MCL) displayed an overexpression of H3K27m3 in the great majority of cases (27/28 (96%) and 14/14 (100%), respectively).

**Fig. 4:**
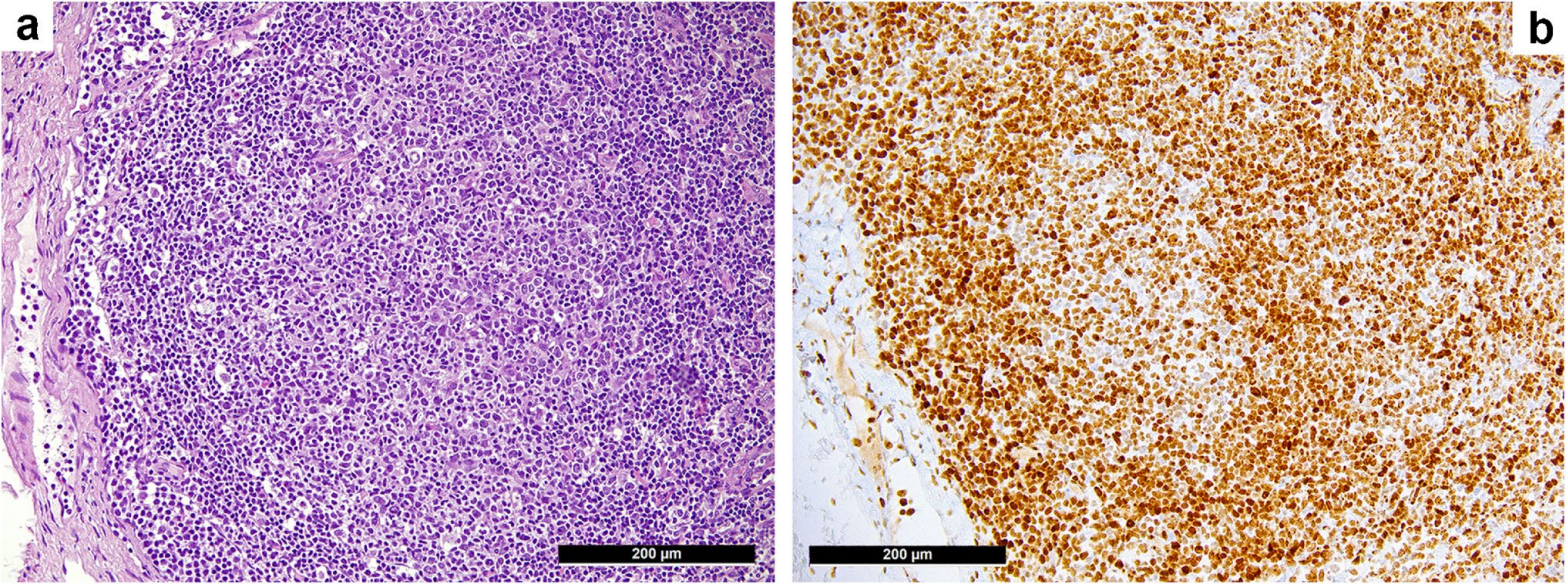
*Unexpected overexpression of H3K27m3 in a supposed case of follicular hyperplasia (FH) of a patient, who developed in follow-up a splenic diffuse red pulp small B cell lymphoma*. H&E staining of an architecturally disturbed germinal center (**a**) and the corresponding striking overexpression of H3K27m3 (**b**) in a supposed FH accompanied by polyclonal plasmacytosis that did not fulfill the diagnostic criteria for a neoplastic process, but revealed several genetic alterations on molecular work-up. Considering the patient’s follow-up, the observed pattern turned out to be most likely an early follicular colonization by a splenic diffuse red pulp small B cell lymphoma that the patient developed later on

Staining for H3K27m3 in the FH cases revealed “physiologic-type” expression results in 40/42 (95%) instances. Only two displayed an overexpression of H3K27m3 in the germinal centers. Therefore, both cases underwent in-depth molecular analysis. The first case (Fig. 4a,b) was neither B or T cell clonal nor contained PCR products of t(14;18) or t(11;14) and did thus not qualify for the diagnosis of a neoplastic process. Thus, the diagnosis of FH accompanied by polyclonal plasmacytosis was rendered. Interestingly, our customized lymphoma panel revealed genetic polymorphisms in 2 different genes (*ATM* M1134L, variant allelic frequency (VAF) 52%, and *KMT2C* S2869C, VAF 55%) as well as one pathogenic *TET2* mutation (R1452*, VAF 19%) and two *NOTCH2* variants of uncertain significance (VUS), S2403C and S2389P, with a VAF 7% each, and turned out being most likely an early follicular colonization by a splenic diffuse red pulp small B cell lymphoma (SDRPL) that the patient developed in the follow-up. Interestingly, the second (also non-clonal) case with conspicuous H3K27m3 expression showed three *KMT2D* VUS (A482E, VAF 7%; L483S, VAF 6%; and H2467P, VAF 43%); unfortunately, the patient was lost from follow-up.

### EZH2 expression

Interpretable results of the EZH2 staining were achieved in 91 FL cases (Table 1, Fig. 2a, b). There was neither a correlation between the expression of EZH2 and H3K27m3 (*P* = 0.373) or BCL2 (*P* = 0.161) nor between EZH2 expression and mutations of its coding gene (Suppl. Table 2).

Eleven FH cases were evaluated, and 5/11 (45%) revealed a moderate and 6/11 (55%) a strong expression of EZH2 (Fig. 2c, d).

## Discussion

Over the last two decades, recurrent somatic mutations of the epigenetic modifier gene *EZH2* have been found to affect the methylation status of H3K27 in DLBCL and FL [5, 8, 10], which, in turn, influences the executing function of H3. In the course of these findings, *EZH2* inhibitors have been developed, representing an emerging group of new therapeutics in patients with recurrent and relapsed DLBCL, and constituting the first biomarker-directed therapy in refractory FL [14, 15, 25, 26]. In order to facilitate the distinction of *EZH2* wild-type from *EZH2* mutant-type DLBCL, the group around Dubois investigated the immunohistochemical expression of EZH2 and mono-, di-, and tri-methylated H3K27 in relation to the *EZH2* somatic mutational status. In the majority of cases, they were able to differentiate patients with wild-type *EZH2* from mutant-type *EZH2* with the help of their ratio of tri- to di-methylated histones (H3K27m3/m2 score), as mutated samples showed a higher expression of H3K27m3 and a lower expression of H3K27m2 [26]. Huet et al. preformed a similar study with FL, proofing that their assessed H3K27m3/m2 score could also differentiate mutated from wild-type samples [9].

In contrast to previous works, the main focus of the present study was to evaluate the expression of H3K27m3 in FL, PCFCL, and PTFL regarding its potential utility in the differential diagnosis to FH and other mature B cell neoplasms.

We found a reliable and constant overexpression of H3K27m3 in the vast majority of FL and in all PCFCL, independent of their grade and, most importantly, independent of both immunohistochemical BCL2 protein expression and *BCL2*-rarrangement-status by FISH. Consequently, the evaluation of H3K27m3 appears to be an easy to perform and inexpensive method that seems to be of high diagnostic value, especially in the setting of discriminating PCFCL from reactive lympho-follicular proliferations in the skin, and BCL2-negative FL from FH. As BCL2-negative FL represents one of the major diagnostic problems, we explicitly enriched our cohort for *BCL2*-rearrangement negative cases (24%). Importantly, these cases did not show different results regarding their H3K27m3 expression. On the opposite, more than 95% of the investigated control cases of FH in our cohort lacked overexpression of H3K27m3 in the germinal centers but showed a distinct physiological pattern and/or zonation. This underlines the diagnostic impact of evaluating the H3K27m3 overexpression in borderline cases between FH and FL or PCFCL, especially when BCL2 immunohistochemistry and/or FISH results are negative or of little diagnostic help. In contrast, PTFL did not overexpress H3K27m3, which fits with the very low proportion of mutations in genes encoding for DNA- and histone-modifying enzymes in general—and *EZH2* in particular—in this entity [3, 23, 28].

Detectable overexpression of H3K27m3 might serve as a caveat in the setting of FH, as one of our two H3K27m3-positive supposed FH that was molecularly worked-up implies. Although not meeting criteria for lymphoma diagnosis (e.g., lack of clonality and translocations), it displayed certain molecular alterations, which were striking for a lymphoid pre-neoplasia. Indeed, during follow-up, the patient was diagnosed with SDRPL. The detected genetic polymorphism in the *KMT2C* gene might have had a certain impact on the observed overexpression of H3K27m3, as KMT2C is at least one of the most important regulators of histone H3K4 methylation [29]. Bearing in mind the later-on diagnosed SDRPL, the two *NOTCH2* VUS with VAF of 7% might have reflected an otherwise non-perceptible infiltration of the respective lymph node, as *NOTCH2* mutations are among the most common genetic alterations in the closely related splenic MZL [30], although the exact identified variants have not been yet described.

The molecular result of our second H3K27m3-positive FH-patient with three *KMT2D* VUS is difficult to explain. One hypothesis might be that its overexpression of H3K27m3 in the germinal centers is a read-out of dysfunctional methylation (being generally characteristic of lymphoid neoplasia), but this, admittedly, is speculation.

Looking at other mature small B cell lymphomas, especially those with possible nodular architecture such as MZL, H3K287m3’s potential as a helpful tool in differential diagnostic considerations is very limited, as the great majority of those entities, namely, MZL, CLL/SLL, and MCL, also display an overexpression of this marker. Merely in the case of MZL, H3K27m3 can help to visualize overrun, non-neoplastic germinal center, as they do not overexpress H3K27m3.

We also investigated the expression of EZH2, as the *EZH2*-status may be predictive in FL since presence of *EZH2* mutations seems to be associated with a better outcome after conventional chemotherapy (R-/O-CHOP/CVP-based), whereas patients without *EZH2* alterations experience longer progression-free survival with R- or O-bendamustine-treatment [31]. Corroborating the lacking predictive potential of anti- EZH2 antibodies regarding the *EZH2* mutational status in FL as well as DLBCL [9, 26], we found the immunohistochemical expression of EZH2 to be non-specific concerning differential diagnostic considerations.

Interestingly and certainly worthy of discussion, H3K27m3 (and EZH2) expression did not correlate with the mutational results of our cohort. For sure, these data need to be treated with caution, as we only analyzed a very limited number of cases. Nevertheless, H3K27m3 overexpression by far extends the proportion of *EZH2* mutant FL in our cohort and in the literature [8, 9]. In our opinion, this might be explained by the fact that the methylation of histones represents the final stretch of epigenetic modification being influenced by much more players than *EZH2*. This hypothesis is supported by data from epithelial tumors: in a large cohort of breast cancers, although subtype-dependent overexpression of EZH2 and H3K27m3 overexpression were found, not a single *EZH2* mutation could be detected [17]. Furthermore, in a recent study on T cell lymphomas, again, no correlation between the immunohistochemical expression of H3K27m3/EZH2 and molecular alterations in the *EZH2* gene could be identified [18]. Whether assessing tri-methylation of H3K27 might help to estimate the therapeutic effect of EZH2 *-*inhibitors remains to be addressed.

 EZH2’s counterpart is the histone demethylase Ubiquitously Transcribed Tetratricopeptide Repeat on chromosome X (UTX) that specifically erases di- and tri-methylation of H3K27 [32], and newer data on DLBCL suspect suppressed UTX to be much more significant than EZH2 with respect to tri-methylation of H3K27 [33]. Unfortunately, as *UTX* is not covered in the applied customized lymphoma panel, our analysis does not cover respective genetic alterations relevant to tri-methylation of H3K27.

To summarize, H3K27m3 is overexpressed in FL and PCFCL, but not in PTFL and FH, and the use of antibodies against H3K27m3 represents an elegant approach to visualize the final result of dysfunctional methylation [34]. The gained knowledge can be easily transferred and applied in routine diagnostics, especially as an additional tool in the differential diagnosis between FL and FH and PCFCL and reactive lympho-follicular proliferations in the skin. However, H3K27m3 does not appear to be helpful in the differential diagnosis with other mature B cell lymphomas, in particular to MZL, since H3K27m3 is overexpressed across all entities. In accordance to this, FH with equivocal H3K27m3 staining results should entail additional molecular and/or clinical work-up in order to exclude early follicular colonization by MZL, as evidenced by one of our cases.

## Supplementary Information

Below is the link to the electronic supplementary material.

Supplementary file1 (DOCX 16 KB)
