# Supplementary material for: H3K27m3 overexpression as a new, BCL2 independent diagnostic tool in follicular and cutaneous follicle center lymphomas
Source: Virchows Arch. 2022 Jun 4;481(3):489–97. doi: 10.1007/s00428-022-03347-y (PMC9485181; doi:10.1007/s00428-022-03347-y)
Supplement: Supplementary file 1 — Supplementary file1 (DOCX 16 KB) [file 428_2022_3347_MOESM1_ESM.pdf]

Supplementary Information, *Virchows Archiv*

H3K27m3 overexpression as a new, BCL2 independent diagnostic tool in follicular and follicle center lymphomas

Magdalena M. Brune, Visar Vela, Susanne Dertinger, Vanessa Borgmann, Stefan Dirnhofer, Alexandar Tzankov*

* Corresponding author:

Prof. Dr. med. Alexandar Tzankov
Institute of Medical Genetics and Pathology
University Hospital Basel
Schoenbeinstrasse 40
CH-4031 Basel
Switzerland
Phone +41 61 265 3229 | Fax +41 61 265 3194
E-Mail: alexandar.tzankov@usb.ch

Suppl. Table 1. Details on the applied antibodies, dilutions and incubation conditions

Antigen	Source	Retrieval	Dilution	Incubation time
**BCL2**				
SP66	Ventana 790-4604	CC1 48	RTU*	12 minutes
E17	Abcam ab32124	CC1 32	RTU	16 minutes
124	Ventana 790-4464	CC1 40	RTU	20 minutes
**H3K27m3**	Cellsignaling cs9733	CC1 64	1:100	24 minutes
**EZH2**	Abcam ab283270	CC1 24	1:100	32 minutes
	*RTU, ready-to-use			

Suppl. Fig. 1 *H3K27m3 expression in follicular lymphoma, low power magnification*

Strong and diffuse overexpression of H3K27m3 in a follicular lymphoma, low grade, contrasting one uninvolved physiologic germinal center in the right upper corner, displaying “physiologic-type” expression.

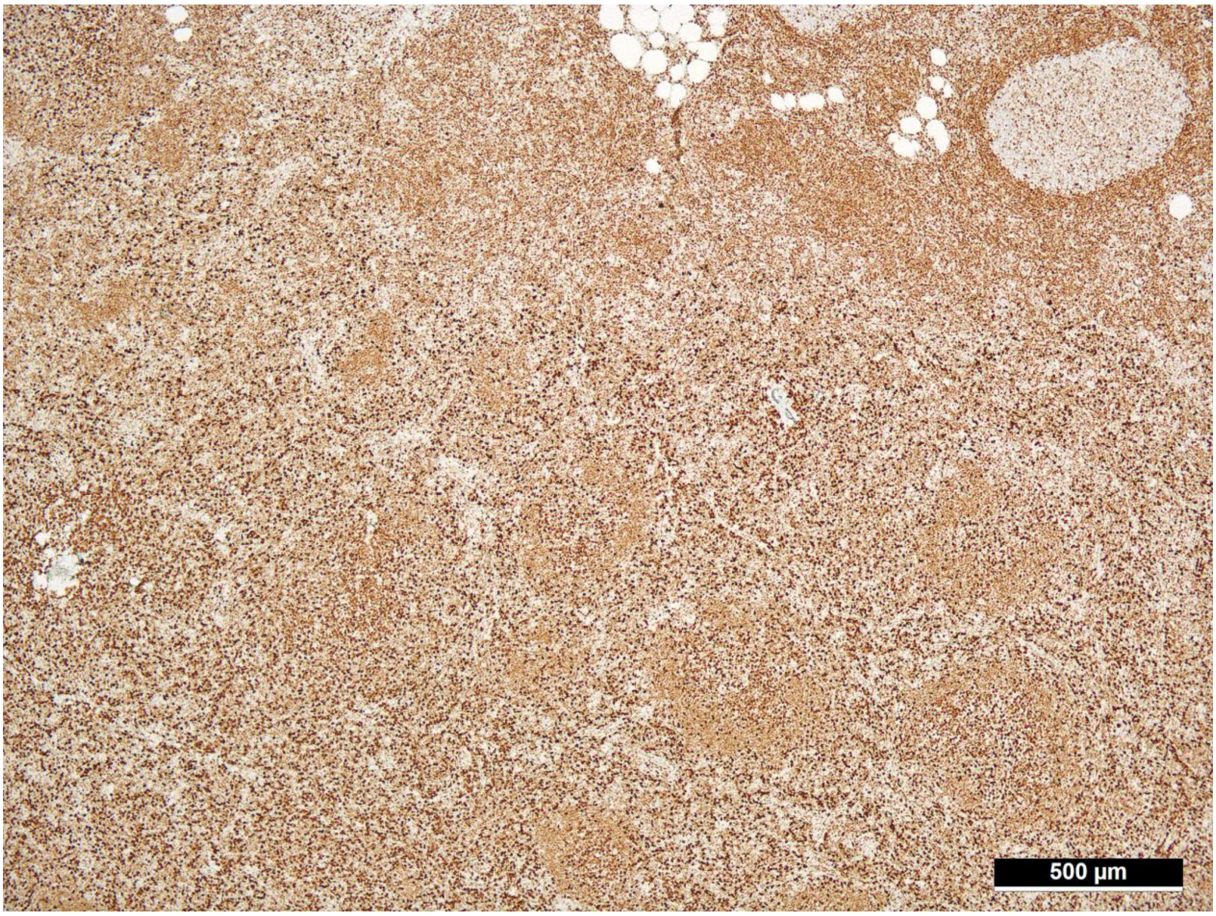

Suppl. Fig. 2 *EZH2 expression in follicular lymphoma, low power magnification*

Partial and weak expression of EZH2 within the same follicular lymphoma, low grade, shown in Suppl. Fig. 1, again contrasting the uninvolved germinal center in the right upper corner, displaying strong EZH2 expression.

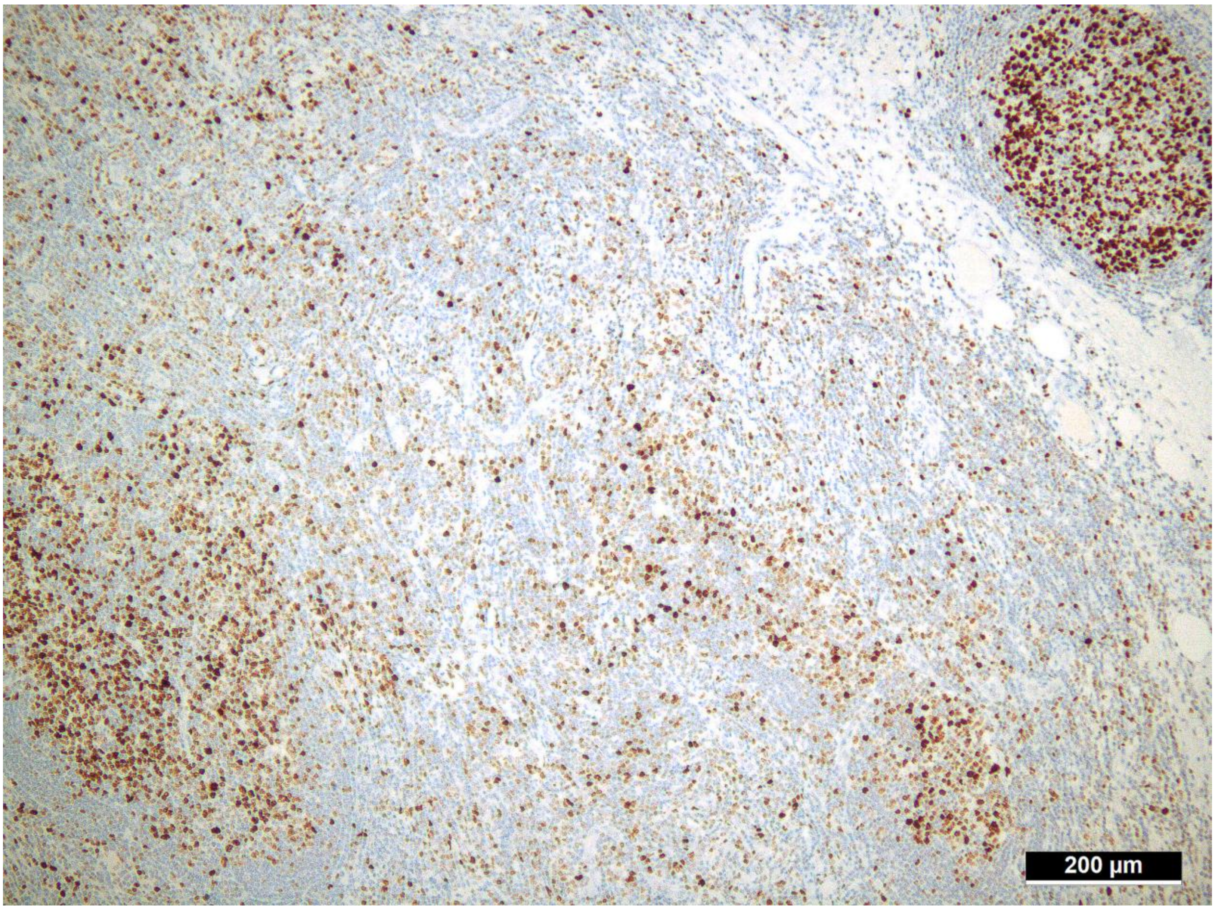

Suppl. Fig. 3 *H3K27m3 expression pattern in marginal zone lymphoma*

Strong and diffuse overexpression of H3K27m3 in a marginal zone lymphoma that colonializes a preexisting rather negative germinal center.

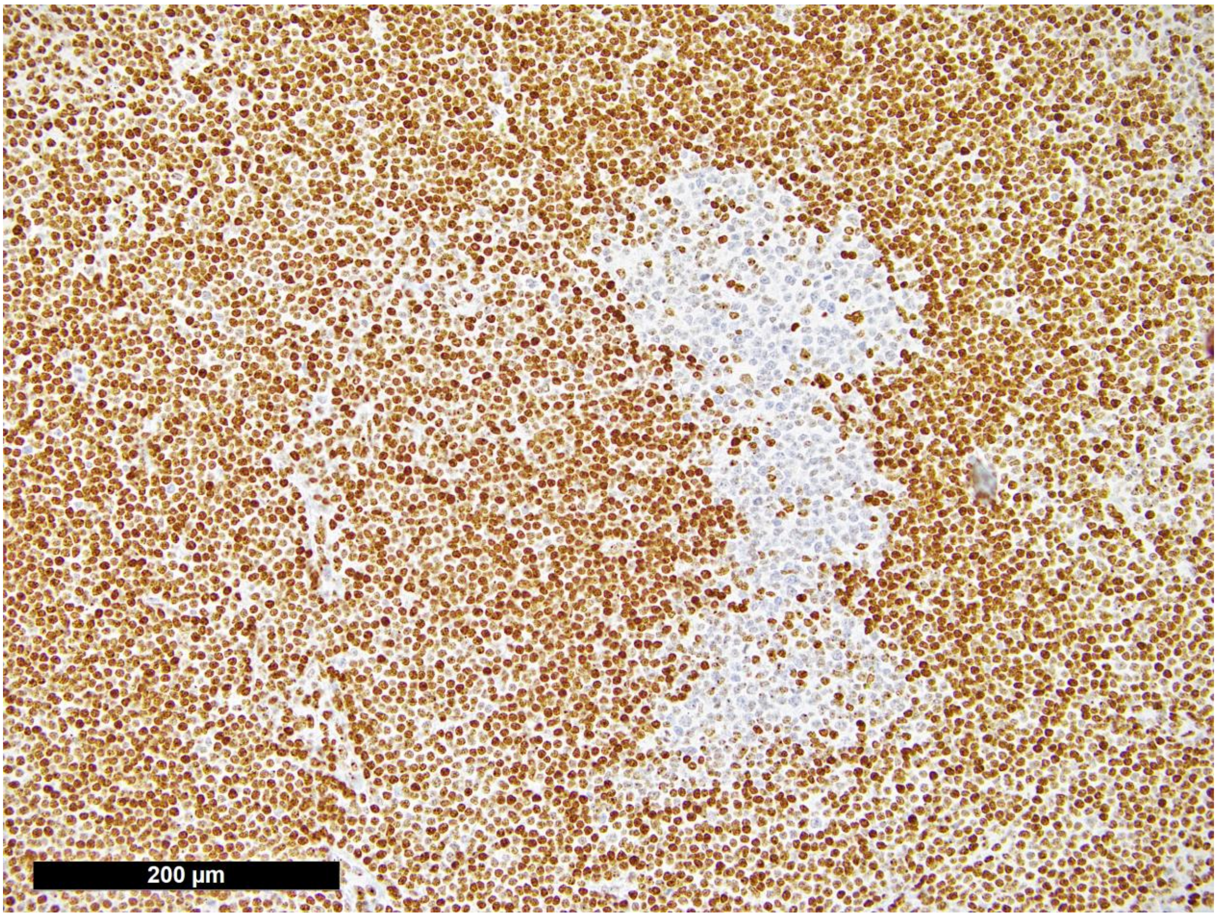

Suppl. Table 2. Molecular analysis of selected cases

Gene	*ATM*	*BCL2*	*CARD11*	*CREBBP*	*EZH2*	*GNA13*	*HIST1H1C*	*KMT2D*	*PIK3CA*	*NOTCH2*	*SF3B1*	*TET2*	*TNFAIP3*
FL (n=14)	1	2	2	7	1	1	1	5	2	1	1	2	1
FH (n=3)	0	0	0	0	0	0	0	0	0	0	0	1	0

14 cases of follicular lymphoma (FL), and 3 cases of follicular hyperplasia (FH) were analyzed with the help of our customized lymphoma panel [18]. Pathogenic or possible/probable pathogenic mutations in 13 out of the 68 examined genes were identified. Here, the absolute numbers of mutant cases per entity is given.

Of the 14 FL cases shown here, only one BCL2-negative (both FISH and immunohistochemically), but *BCL6*-rerranged FL showed a “physiologic type” H3K27m3 expression, while all others stained diffusely and intensively for H3K27m3. Mutation analysis revealed 2 *KMT2D* mutations Q2932fs [variant allelic frequency (VAF) 12%] and L1953fs (VAF 18%)] as well as one *NOTCH2* mutation [R2400*, VAF 19%]. Interestingly, this case also displayed a moderate expression of EZH2, which was not found in the other examined cases showing a lacking or only weak EZH2 expression.

One of the 3 NGS-sequenced FH cases had no detectable pathogenic or possible/probable pathogenic mutations and a “physiologic type” expression of H3K27m3. The other case, that displays the *TET2* mutation, is the extensively discussed FH case with H3K27m3 overexpression and genetic polymorphisms in *ATM* and *KMT2C* as well as variants of uncertain significance in *NOTCH2.* The third displayed three *KMT2D* VUS and also overexpressed H3K27m3. EZH2 has not been analyzed.
